# RETRACTION: Aberrant KIF20A Expression Is Associated with Adverse Clinical Outcome and Promotes Tumor Progression in Prostate Cancer

**DOI:** 10.1155/dim/9816547

**Published:** 2026-10-26

**Authors:** Disease Markers

RETRACTION: Z. Zhang, C. Chai, T. Shen, et al., “Aberrant KIF20A Expression Is Associated with Adverse Clinical Outcome and Promotes Tumor Progression in Prostate Cancer,” *Disease Markers*, 2019, 4782730, https://doi.org/10.1155/2019/4782730.

The above article, published online on 3 September 2019 in Wiley Online Library (wileyonlinelibrary.com), has been retracted by John Wiley & Sons Ltd. The retraction has been agreed due to multiple concerns identified with the figures. Initially, concerns pertaining to Figure 2 were reported on PubPeer [1], after which the authors contacted the journal for correction. However, subsequent investigation identified additional concerns with Figures 2 and 3. Specifically:

• In Figure 2e:
  ◦ The shCON Migration panel is duplicated in the shCON invasion panel, having been rotated 84 degrees
  ◦ The shKIF20A migration panel is duplicated in the shKIF20A panel
• In Figure 2f:
  ◦ The PCNA bands are identical to the KIF20A bands shown in Figure 3c
  ◦ The Ki67 bands in Figure 2f are highly similar to the KIF18A bands shown in Figure 2a of [2], a previous publication with shared authors

After assessment of the concerns and the author’s responses, the results and conclusions can no longer be considered reliable, and the article is therefore retracted.

The authors disagree with the retraction.
